# *Melissa officinalis* Regulates Lipopolysaccharide-Induced BV2 Microglial Activation via MAPK and Nrf2 Signaling

**DOI:** 10.4014/jmb.2409.09020

**Published:** 2024-10-29

**Authors:** Ji-Won Choi, Sang Yoon Choi, Guijae Yoo, Ho-Young Park, In-Wook Choi, Jinyoung Hur

**Affiliations:** 1 Korea Food Research Institute, Wanju-Gun, Jeollabuk-do 55365, Republic of Korea; 2 Division of Food Biotechnology, University of Science and Technology, Daejeon 34113, Republic of Korea

**Keywords:** *Melissa officinalis*, neuroinflammation, neurodegenerative disease, BV2 microglia, TLR4, Nrf2

## Abstract

Neuroinflammation and microglial activation play critical roles in neurodegenerative diseases such as Alzheimer's and Parkinson's disease. Modulating microglial activation may help prevent the progression of these disorders. This study aimed to investigate the effects and mechanisms of *Melissa officinalis* ethanol extract on lipopolysaccharide (LPS)-induced microglial activation in BV2 cells. Cell viability and nitric oxide (NO) production were assessed using MTT assay and Griess reagent, while inflammatory cytokine levels were measured by qPCR. Key inflammatory pathways, including MAPK, TLR4, and antioxidant biomarkers, were analyzed through western blot and immunofluorescence. Rosmarinic acid content in *M. officinalis* was determined using high-performance liquid chromatography (HPLC). The results demonstrated that *M. officinalis* ethanol extract significantly inhibited LPS-induced NO production and reduced inflammatory cytokine expression. Additionally, it downregulated inducible nitric oxide synthase (iNOS), cyclooxygenase-2 (COX-2), TLR4, NF-κB, and MAPK signaling pathways (p38, JNK, ERK), while increasing the expression of antioxidant markers, including Nrf2, HO-1, catalase, and SOD2. In conclusion, *M. officinalis* ethanol extract exerts neuroprotective effects by modulating inflammation and enhancing antioxidant defenses, suggesting its potential in the prevention and treatment of inflammation-related neurodegenerative diseases.

## Introduction

Neuroinflammation is increasingly recognized as a pivotal factor in the progression of several neurodegenerative diseases, including Alzheimer’s disease, Parkinson’s disease, and amyotrophic lateral sclerosis (ALS) [1, 2]. A key component of neuroinflammation is the activation of glial cells, particularly microglia and astrocytes, which can exacerbate neuronal damage through chronic inflammation and oxidative stress[3, 4]. Microglia, the resident immune cells of the central nervous system (CNS), play a dual role—initially protecting neurons, but when overactivated during prolonged inflammation, they contribute to neurodegeneration by producing reactive oxygen species (ROS) and inflammatory cytokines [5, 6]. Excessive microglial activation can thus result in neuronal injury, furthering the progression of diseases such as Alzheimer’s and Parkinson’s [7].

Toll-like receptor 4 (TLR4), expressed on microglial surfaces, plays a crucial role in modulating innate and adaptive immune responses by recognizing pathogen-associated molecular patterns like lipopolysaccharide (LPS) [8]. LPS, a key component of Gram-negative bacterial walls, binds to TLR4 and activates downstream signaling pathways, including the mitogen-activated protein kinase (MAPK) cascade and nuclear factor kappa B (NF-κB). This leads to the production of pro-inflammatory cytokines such as interleukin-1β (IL-1β), IL-6, and tumor necrosis factor-alpha (TNF-α), which exacerbate neuroinflammation[8-10]. Furthermore, LPS-induced activation increases inducible nitric oxide synthase (iNOS) and cyclooxygenase-2 (COX-2), enhancing nitric oxide (NO) production and ROS levels, contributing to neurodegenerative damage [11, 12].

The receptor for advanced glycation end products (RAGE), another significant player in neuroinflammation, is a multi-ligand receptor that binds to LPS, advanced glycation end products (AGEs), and other molecules. Upon activation, RAGE promotes the generation of ROS and further activates NF-κB, aggravating inflammatory responses in the CNS [13, 14].

*M. officinalis*, or lemon balm, is a perennial herb from the Lamiaceae family, known for its calming effects and traditional use in treating stress, anxiety, and sleep disorders. Recent studies have explored its pharmacological potential, revealing antioxidant, antimicrobial, antiviral, and cardioprotective properties, attributed to bioactive compounds such as rosmarinic acid, flavonoids, and essential oils like citral, citronellal, and geraniol [15-18]. Rosmarinic acid, in particular, demonstrates strong antioxidant activity by scavenging free radicals and regulating inflammatory pathways [19, 20]. Despite evidence of its neuroprotective effects, there is limited research on its role in modulating microglial activation. This study aims to investigate *M. officinalis*’s potential to regulate microglial activity and inflammatory responses, highlighting its possible therapeutic application in neuroinflammatory and neurodegenerative disorders. This study investigated the anti-inflammatory effect of lemon balm ethanol extract on microglia. LPS was used to activate microglia and induce inflammation, and the efficacy of lemon balm extract was evaluated by analyzing inflammatory pathways, such as MAPK and NF-κB pathways. Moreover, the antioxidant activity of lemon balm was investigated by measuring ROS.

## Materials and Methods

Dulbecco’s modified eagle medium (DMEM), fetal bovine serum (FBS), and penicillin-streptomycin (PS) were purchased from Gibco (USA). LPS, Griess reagent, 3-(4,5-dimethyl-2-thiazolyl)-2,5-diphenyl tetrazolium bromide (MTT) powder, and dimethyl sulfoxide (DMSO) were acquired from Sigma Chemical Company (USA). RNA purification kit was purchased from MACHEREY-NAGEL (Neumann, Germany). Primary antibodies and secondary antibodies used for western blot analysis were purchased from Cell Signaling Technology (USA) and Santa Cruz Biotechnology (USA).

### Preparation of *M. officinalis* Extract (MOE)

After drying the leaves of *M. officinalis* (KFRI-GAP-0079, Republic of Korea) was extracted twice with 70%ethanol at 50°C for 3 h. The extracted solution was filtered, evaporated, and concentrated, and lyophilized using a freeze-dryer. The powdered product was stored at −20°C. The yield of the *M. officinalis* ethanol extract was 21.3%, based on dry weight.

### Quantitative Analysis by High-Performance Liquid Chromatography (HPLC)

*M. officinalis* leaf extract powder was dissolved in MeOH (10 mg/ml) and filtered through a syringe filter (0.2 μm) to prepare the sample stock solutions. A standard stock solution of rosmarinic acid was also prepared by dissolving 1 mg rosmarinic acid in 1 ml MeOH. HPLC analysis was performed on a Waters e2695 Separations Module with 2998 Photodiode Array detector. Separation was obtained by gradient elution on a Waters XBridge BEH C_18_ column (3.5 μm, 4.6 × 150 mm, 130Å) at 23°C. The mobile phase was (a) 0.1% formic acid in water and (b) 0.1% formic acid in acetonitrile and delivered at 1.0 ml/min according to the following linear gradient: 0–30 min, 5–35% B; 30–35 min, 35–100% B; 35–37 min, 100% B; 37–37.1 min, 100–5% B; 37.1–40 min, 5% B. The injection volume was 5 μl and monitored at 320 nm.

### Cell Culture and Viability Assay

Murine microglial BV2 cells, kindly provided by Dr. Myungsook Oh (Kyunghee University, Republic of Korea), were cultured in DMEM supplemented with 10% heat-inactivated fetal bovine serum and 1% PS in a humidified incubator at 37°C with 5% CO_2_. BV2 cells were seeded into 96-well plates at a density of 5 × 10^4^ cells per well in 100 μl of medium and treated with LPS (0.1 μg/ml) in the presence or absence of MOE at concentrations of 10, 50, and 100 μg/ml for 18 h. Following treatment, the medium was replaced with MTT solution, and cells were incubated for 4 h at 37°C. The resulting formazan crystals were dissolved in 100 μl of DMSO, and absorbance was measured at 595 nm using a microplate reader (Molecular Devices, USA).

### Determination of NO Production

BV2 cells were plated into 96-well plates (5 × 10^4^ cells/well) and treated with LPS (0.1 μg/ml) and MOE (10, 50, and 100 μg/ml) for 18 h. To indirectly assay LPS-mediated NO production, culture supernatants (50 μl per sample) were mixed with equivalent volumes of Griess reagent and incubated for 10 min at 23°C. The absorbance values were measured at 560 nm using a microplate reader, and NaNO_2_ was used as the standard to determine NO_2_ concentrations.

### Real-Time Polymerase Chain Reaction (RT-PCR)

Total RNA was isolated from cells using NucleoSpin RNA and synthesized into complementary DNA using the iScript cDNA Synthesis kit. RT-PCR was performed using an iQ SYBR Green Supermix kit (Bio-Rad, USA). The primer sequences used were TNF-α (NM_013693), forward (5'-CCCTCACACTCAGATCATCTTCT-3') and reverse (5'-GCTACGACGTGGGCTACAG-3'); IL-1β (NM_008361), forward (5'-GAAATGCCACCTTTTGAC AGTTG-3') and reverse (5'-TGGATGCTCTCATCAGGACAG-3'); IL-6 (NM_031168), forward (5'-TAGTCC TTCCTACCCCAATTTCC-3') and reverse (5'-TTGGTCCTTAGCCACTCCTTC-3'); NLRP3 (NM_145827) forward (5'-ATTACCCGCCCGAGAAAGG-3') and reverse (5'-TCGCAGCAAAGATCCACACAG-3'); and GAPDH (NM_008084), forward (5'-CTGACTTCAACAGCGACACC-3') and reverse (5'-TGCTGTAGCCAA ATTCGTTGT-3').

### Cytokine Determination by Enzyme-Linked lmmunosorbent Assay (ELISA)

After treatment with LPS and MOE culture supernatants were collected to measure the levels of pro-inflammatory cytokines, including TNF-α and IL-6. The concentrations of cytokines were determined using specific ELISA kits: TNF-α was measured using an ELISA kit from R&D Systems (USA), and IL-6 was measured using an ELISA kit from Invitrogen (USA), following the manufacturers’ instructions.

### Western Blotting

Cells were lysed with the PRO-PREP protein extraction solution (iNtRON Biotech, USA). Nuclear and cytosol fractions from treated BV2 cells were extracted using the nuclear/cytosol kit (Thermo Fisher Scientific, USA). Proteins were separated by 10% SDS-PAGE and transferred onto PVDF membranes. The membranes were blocked with 5% skim milk and then incubated overnight at 4°C with primary antibodies: iNOS, COX-2, TLR4, p38, phospho (p)-p38, ERK, p-ERK, JNK, p-JNK, p65, p-p65, GLO1, RAGE, Catalase, SOD2, Lamin B1, Nrf2, HO-1, and β-actin (Cell Signaling Technology). After washing, the membranes were incubated with secondary antibodies (Invitrogen) for 1 h at 23°C. Protein bands were then measured using the ChemiDoc XRS + imaging system (Bio-Rad). Band intensities were quantified to β-actin or Lamin B1 band using the Image Lab Software (Bio-Rad).

### Immunofluorescence

Cells were seeded in a cell culture 4-chamber slide (1 × 10^5^ cells/well) for 24 h, and then treated with various concentrations of MOE (10, 50, and 100 μg/ml) for 18 h. The cells were fixed with 4% formaldehyde for 15 min, permeabilized with 0.05% saponin in PBS for 30 min, and blocked with 1% BSA (Bovine Serum Albumin) in PBS for 30 min at 23°C. Cells were incubated with Nrf2 antibodies (1:200 in 1% BSA in PBS) overnight at 4°C and then with Invitrogen Alexa Fluor Plus 488 goat anti-mouse IgG secondary antibodies (1:500) for 1 h at 23°C. Images were taken on an FV3000 confocal system microscope (Olympus, Japan) at ×20 using the FLUOVIEW software. The image contained an overlay of cytosol (green) and nuclei (blue).

### Statistical analysis

Data are presented as mean ± standard error of the mean. Comparisons between groups were performed using one-way ANOVA followed by Tukey’s test. Statistical significance was set at *p* < 0.05. All data were analyzed using GraphPad Prism 9 (GraphPad Software, Inc., USA).

## Results

### Quantitative Analysis of the Major Compound

**Fig. 1:**
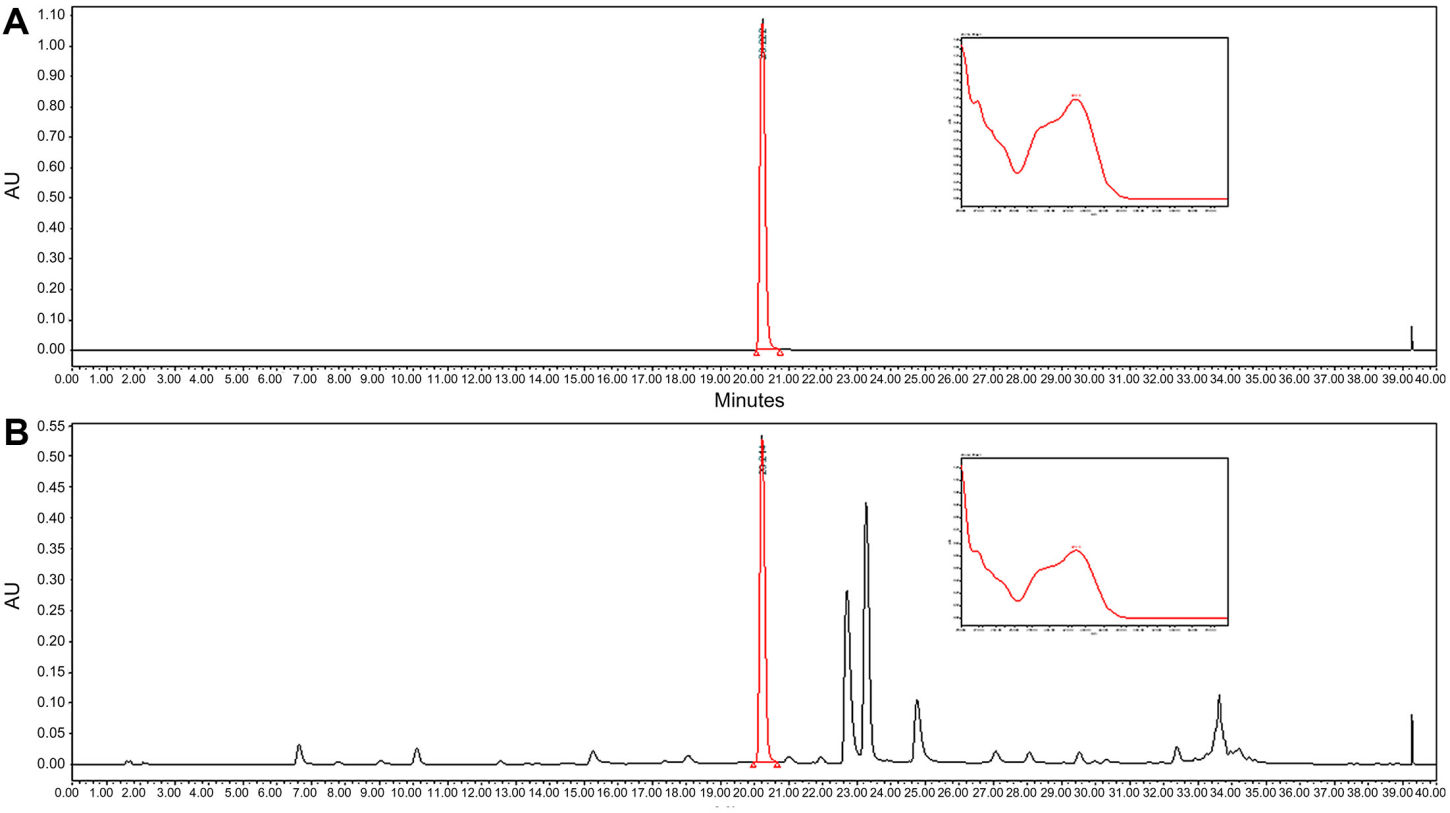
The high-performance liquid chromatography chromatogram of rosmarinic acid (A) and *M. officinalis* extracts (B).

The major compound in MOE was identified by comparing the retention time and UV spectra with the standard compound. The linearity of the calibration curve was determined based on the correlation coefficient (R^2^), and the rosmarinic acid concentrations in the MOE were then calculated from the calibration curve of the standard compound. The content of the major compound rosmarinic acid was 45.5 ± 0.7 mg/g. Fig. 1 shows representative HPLC chromatograms of the MOE and standard solutions. The limit of detections and quantifications for the standard compound are summarized in Table 1. The intra- and inter-day variations of rosmarinic acid were assessed by MOE analysis (Tables 2 and 3).

### Effects of MOE on Cell Viability, NO Production, iNOS and COX-2 Protein Expression in LPS-Stimulated BV2 Microglial Cells

**Fig. 2:**
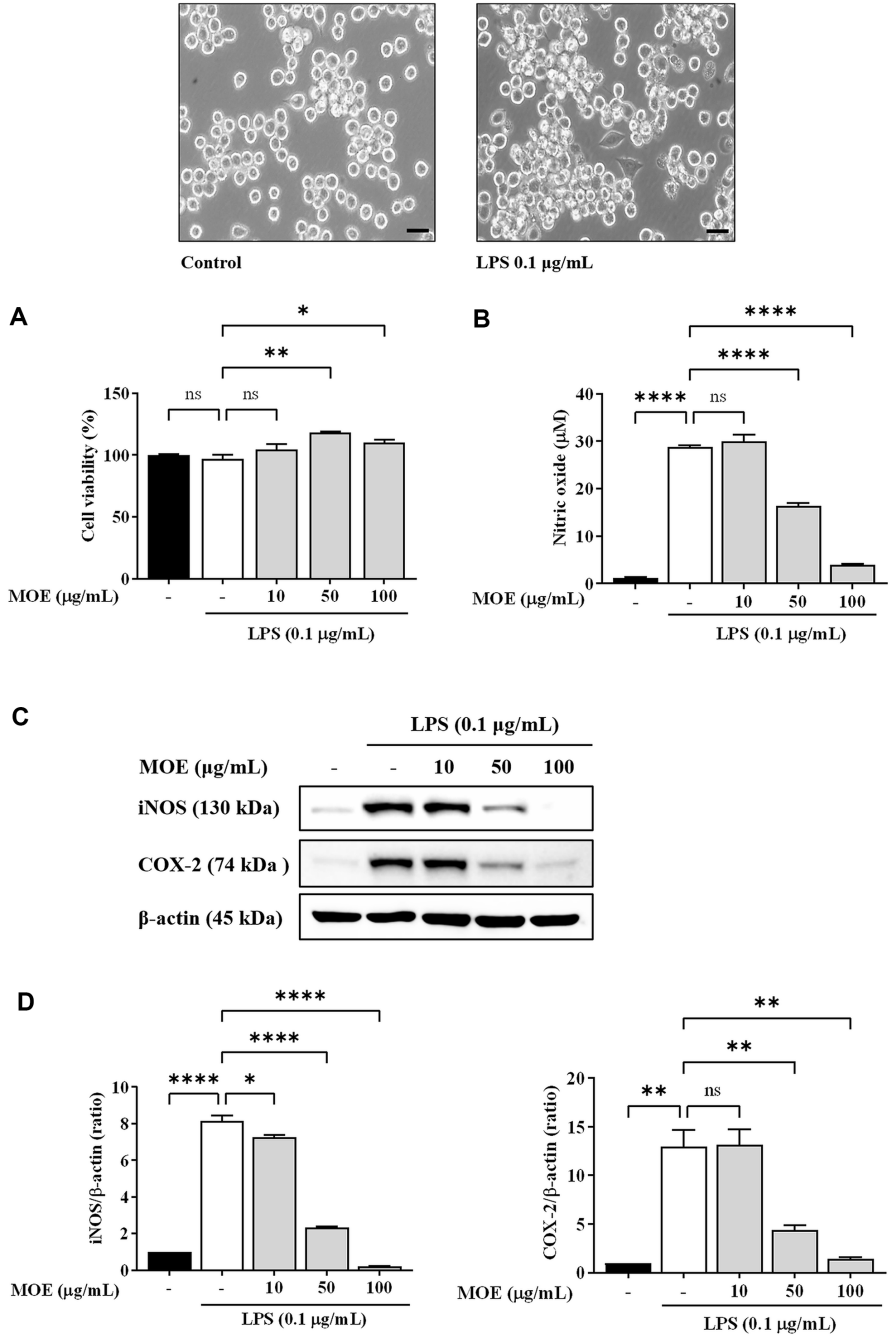
Effects of MOE on NO and COX-2 inhibition in BV2 microglia. (**A**) Cell viability was evaluated using the MTT method. (**B**) Measurement of NO using Griess reagent. (**C, D**) Effects of MOE on the protein expression of iNOS and COX-2, as detected by western blotting. Results are expressed as mean ± SEM of three independent experiments. *p < 0.05, **p < 0.01, ***p < 0.01, ****p < 0.01 vs LPS-treated group.

The MTT assay showed that cell viability was not affected at any concentration (10, 50, and 100 μg/ml) used (Fig. 2A), confirming that the inhibition of NO production in LPS-induced BV2 cells was not due to the cytotoxic effects of MOE. Moreover, NO production was significantly enhanced in the LPS-treated group compared to the control group (from 1.1 μM to 28 μM; Fig. 2B). However, MOE treatment significantly suppressed LPS-induced NO production in a concentration-dependent manner. In particular, treatment with 100 μg/ml MOE markedly inhibited NO overproduction from 28.0 μM to 4.0 μM.

We measured the protein levels using western blotting (Fig. 2C). iNOS and COX-2 expression increased by approximately 8.1 and 12.9 fold, respectively, in the LPS-treated group compared to the control group. MOE (10, 50, and 100 μg/ml) reduced iNOS expression in a dose-dependent manner to 7.2, 2.3, and 0.2 fold, respectively, in the LPS-treated group compared to the control group (1.0 μM). MOE (50 and 100 μg/ml) markedly reduced COX-2 expression; however, MOE (10 μg/ml) did not reduce COX-2 expression (Fig. 2D).

### MOE Attenuated Pro-Inflammatory Cytokine Expression in LPS-Stimulated BV2 Microglia

**Fig. 3:**
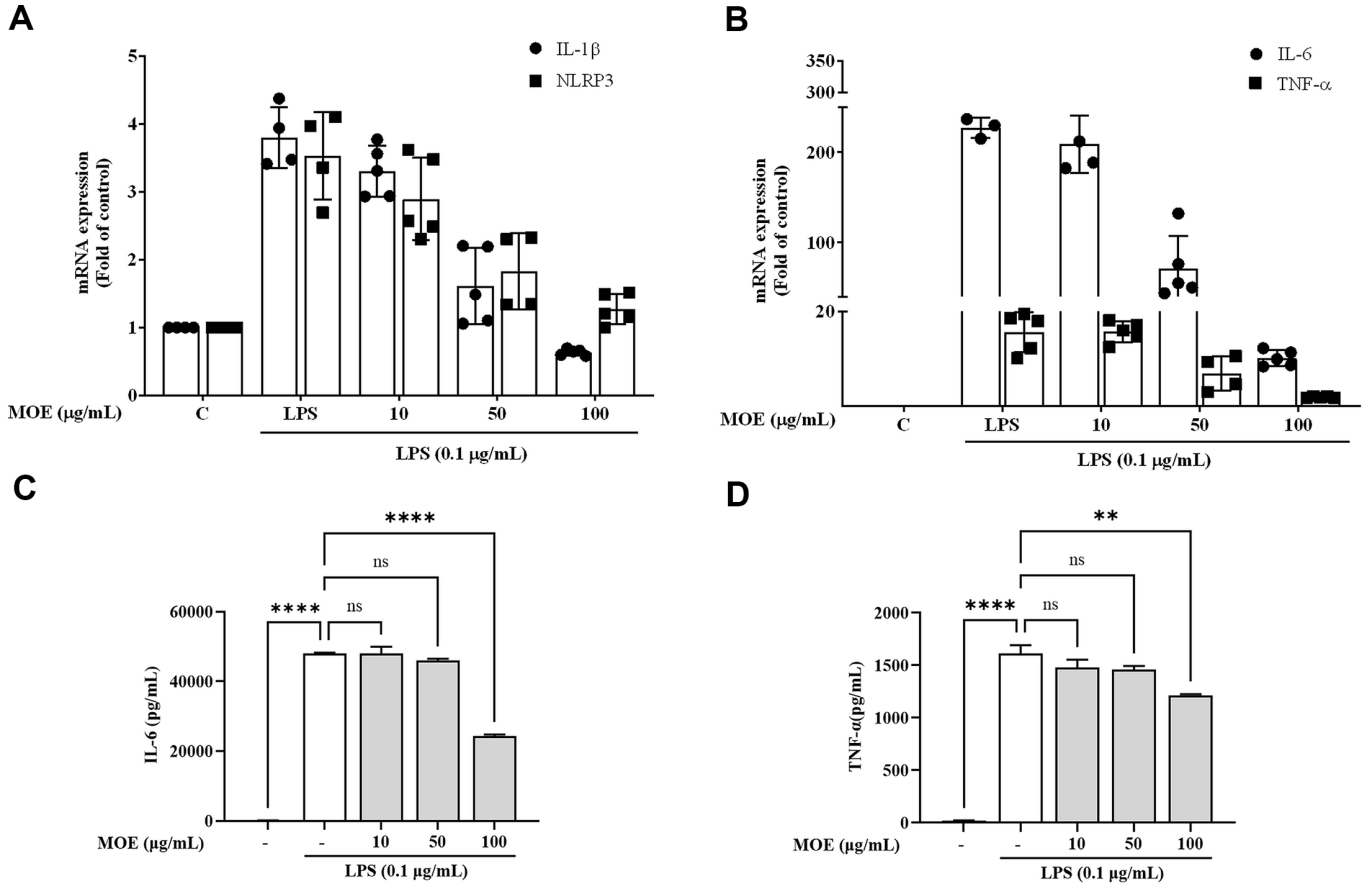
Effects of MOE on the mRNA expression of inflammatory cytokines (IL-1β, IL-6, TNF-α, and NLRP3) (A, B) and protein expression of (IL-6 and TNF-α) (C, D). The mRNA expression of IL-1β, NLRP3, TNF-α, and IL-6 were analyzed by RT-PCR and protein expressions were conducted by ELISA. Results are expressed as mean ± SEM of three independent experiments. ** *p* < 0.01, ****p < 0.01 vs LPS-treated group.

The gene expression of IL-1β, IL-6, TNF-α, and NLRP3 were increased by 4.0, 227.0, 16.0, and 3.5 times, respectively, in LPS-treated BV2 cells compared to the control group (Fig. 3). IL-1β gene expression was lower in the LPS and MOE 100 μg/ml treatment group than in the control group (Fig. 3A). IL-6 and TNF-α gene expression were significantly reduced when MOE and LPS were co-administered than when LPS alone was administered (Fig. 3B). Furthermore, NLRP3 gene expression was increased by LPS; however, MOE and LPS co-treatment showed a concentration-dependent decrease in its gene expression (Fig. 3A). we also confirmed the protein expression of pro-inflammatory cytokines using ELISA. The IL-6 and TNF-α protein level increased by LPS and decreased at 100 μg/ml of MOE (Fig. 3C and 3D). Therefore, MOE suppressed LPS-induced gene expression of cytokines in BV2 cells, suggesting that MOE could suppress inflammatory response through the inhibition of cytokine, iNOS, and COX-2 expression.

### Inhibitory Effects of MOE on the Activation of MAPKs and NF-κB in LPS-Stimulated BV2 Microglia

**Fig. 4:**
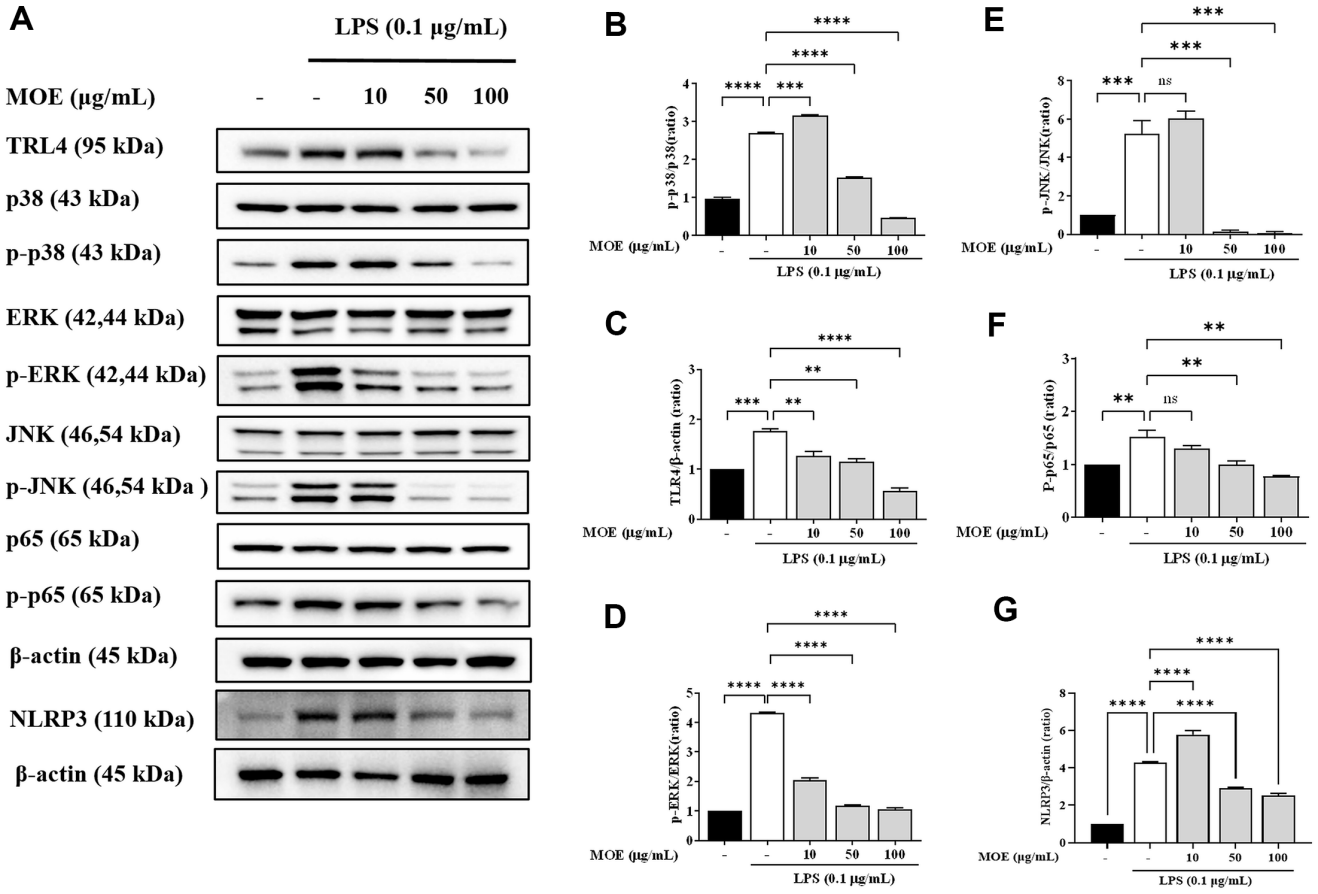
Effects of MOE on the protein expression of TLR4, p38, ERK, JNK, p65 and NLRP3. The expression of TLR4, p38, phosphorylated p38, ERK, phosphorylated ERK, JNK, phosphorylated JNK, p65, and phosphorylated p65 were detected by western blotting. Results are expressed as mean ± SEM of three independent experiments. **p < 0.01, ***p < 0.01, ****p < 0.01 vs LPS-treated group.

Protein expression of p38, ERK, and JNK phosphorylation in the LPS-induced BV2 cells were increased by 3.1, 4.3, and 5.2 times, respectively, compared to the control group (Fig. 4). LPS and MOE 10 μg/ml co-treatment did not significantly change p38 and JNK protein expression; however, LPS and MOE 100 μg/ml co-treatment decreased their protein expression compared to the control group. Particularly, 50 and 100 μg/ml MOE-treated groups significantly reduced JNK expression than the control group. Moreover, ERK protein expression was significantly reduced in the MOE (10, 50, and 100 μg/ml) and LPS co-treated group compared to the LPS-only treated group. In BV2 cells, LPS increased NF-κB activity by 1.5 times. However, NF-κB expression was significantly lowered by LPS and MOE co-treatment. NLRP3 protein expression also Collectively, MOE inhibited NF-κB activity and MAPK phosphorylation, an upstream signaling pathway of NF-κB activation. NLRP3 protein expression was assessed via western blot analysis. LPS treatment resulted in a 4.3-fold increase in NLRP3 protein expression, whereas MOE (100 μg/ml) significantly reduced it by 2.5-fold (Fig. 4).

### Effects of MOE on GLO1 and RAGE Expression

**Fig. 5:**
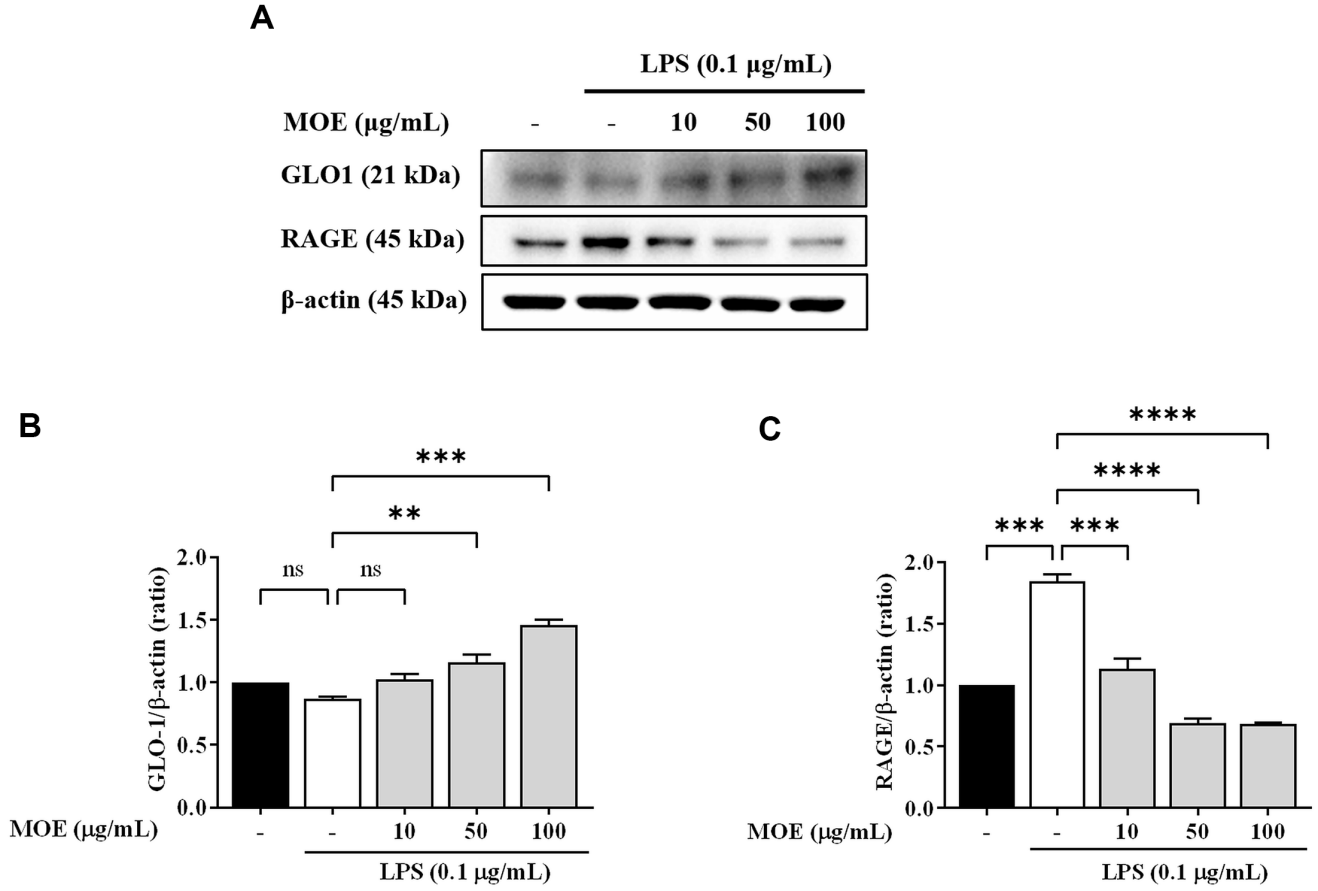
Effects of MOE on the protein expression of GLO1 and RAGE. The expression of GLO-1 and RAGE were detected by western blotting. Results are expressed as mean ± SEM of three independent experiments. **p < 0.01, ***p < 0.01, ****p < 0.01 vs LPS-treated group.

LPS did not significantly decrease GLO1 activation, but 50 and 100 μg/ml MOE increased GLO1 activity (Fig. 5). However, LPS increased RAGE expression in BV2 cells by 1.8 times. The LPS and MOE (50 μg/ml)-treated group decreased LPS-induced RAGE expression by more than 60%. Therefore, MOE increased GLO1 activity but decreased LPS-induced RAGE expression.

### The Effect of MOE on Antioxidant Protein Expression in BV2 Microglia

**Fig. 6:**
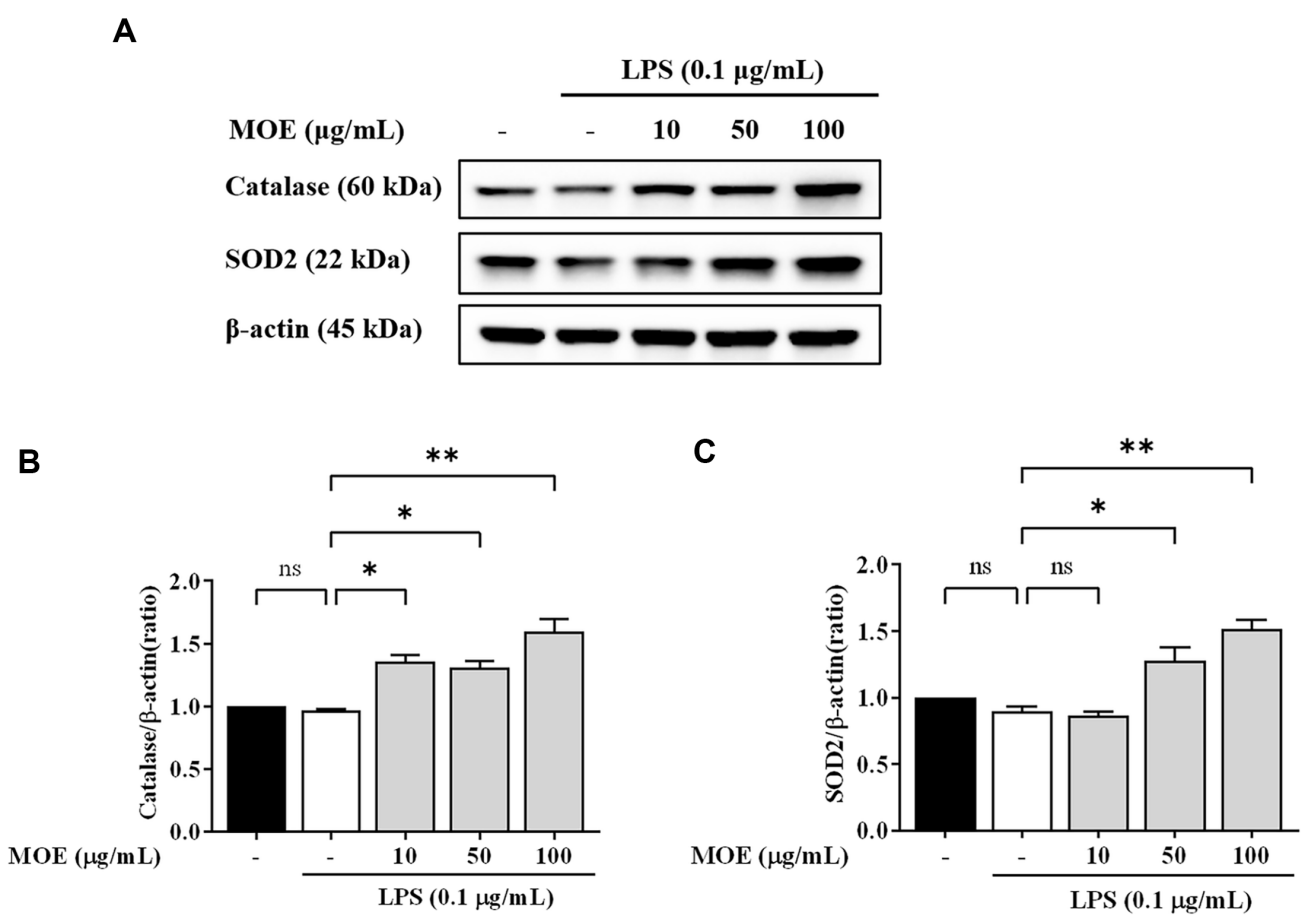
Effects of MOE on the expression of Catalase and SOD2 antioxidant proteins. The expression of catalase and SOD2 were detected by western blotting. Results are expressed as mean ± SEM of three independent experiments. *p < 0.05, **p < 0.01 vs LPS-treated group.

LPS did not change catalase and SOD2 gene expression in BV2 cells (Fig. 6). However, LPS and 100 μg/ml MOE co-treatment increased catalase protein expression by more than 1.5 times compared to the control group. LPS and MOE at 50 and 100 μg/ml increased SOD2 protein expression by 1.3 and 1.5 times, respectively, compared to the control group, suggesting that MOE activated antioxidant enzymes, such as catalase and SOD2, in BV2 cells, thereby protecting the cells.

### MOE Activated Nrf2 Expression and Translocation in BV2 Microglia

**Fig. 7:**
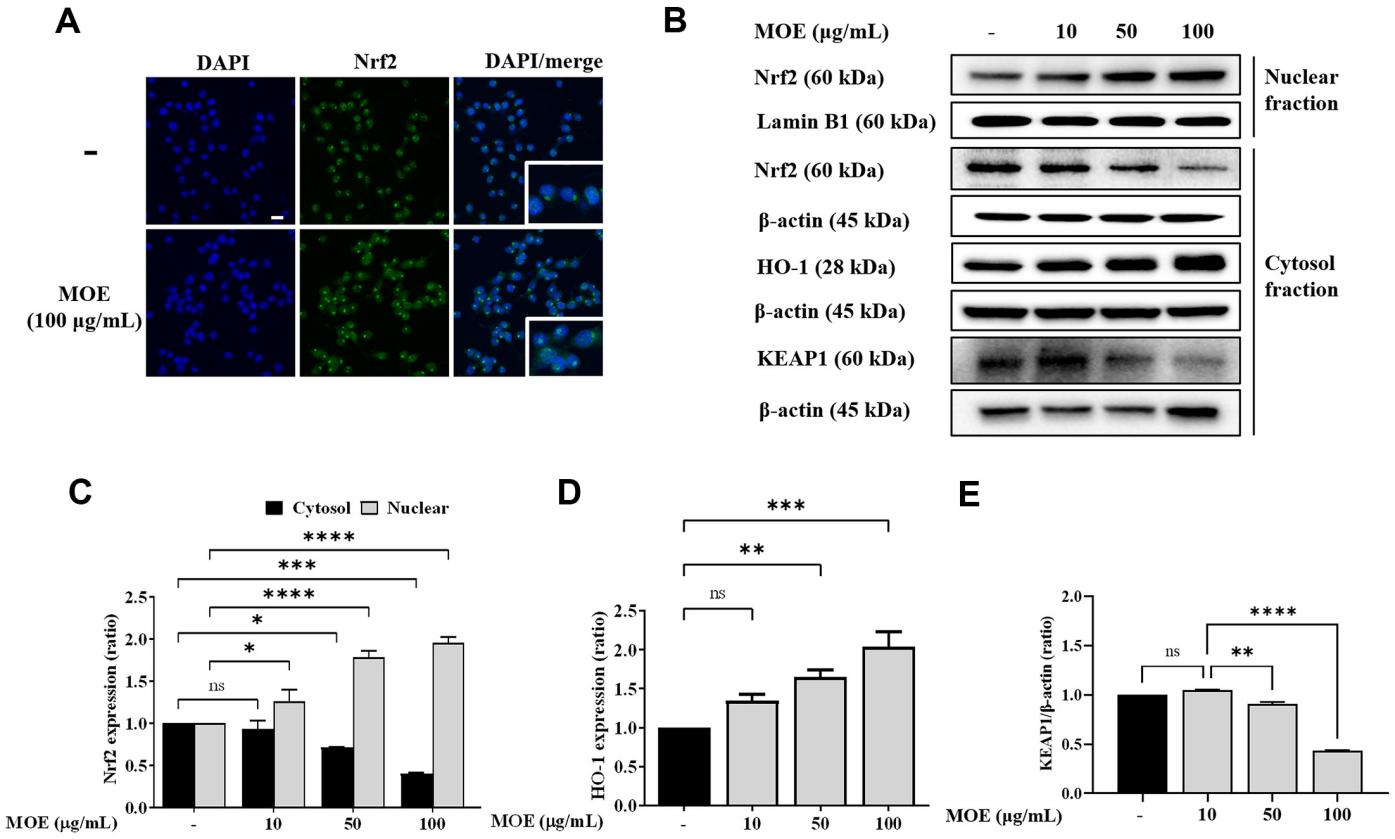
Effects of MOE on Nrf2 nuclear translocation (A) and Nrf2, HO-1 and KEAP1 protein expression (B-E) Representative immunofluorescent images showing nuclei (blue) with Nrf2 (green) in BV2 cells. (**A**) Protein expression of Nrf2, HO-1 and KEAP1 was normalized to that of β-actin in cytosolic fractions and to that of lamin B in nuclear fractions. Results are expressed as mean ± SEM of three independent experiments. *p < 0.05, **p < 0.01, ***p < 0.01, ****p < 0.01 vs Control group. Olympus FluoView 300, ×40, Scale bar 30 μm.

In the MOE-treated group, Nrf2 expression was significantly decreased in the cytoplasm, however, significantly increased in the nucleus (Fig. 7B and 7C), indicating that Nrf2 was translocated into the nucleus by MOE. These results were consistent with those of immunostaining (Fig. 7A). Contrarily, MOE increased cytoplasmic HO-1 and KEAP1 protein expression, suggesting that MOE enhanced the nuclear translocation of Nrf2, which subsequently led to increased HO-1 expression (Fig. 7C-7E).

## Discussion

The findings from this study provide compelling evidence that *M. officinalis* extract (MOE) exerts significant anti-inflammatory and antioxidant effects in LPS-stimulated BV2 microglial cells. These effects are mediated through the modulation of key signaling pathways involved in neuroinflammation, including the MAPK and NF-κB pathways, as well as the enhancement of antioxidant defense systems. The results suggest that MOE could be a promising therapeutic agent for managing neuroinflammatory and neurodegenerative diseases characterized by excessive microglial activation and oxidative stress.

Excessive microglial activation plays a critical role in the progression of neurodegenerative diseases such as Alzheimer's disease, Parkinson's disease, and amyotrophic lateral sclerosis (ALS), primarily through the overproduction of pro-inflammatory cytokines and reactive oxygen species (ROS) [15, 16]. In this study, LPS stimulation significantly increased the production of pro-inflammatory cytokines (IL-1β, IL-6, and TNF-α), nitric oxide (NO), and the expression of inducible nitric oxide synthase (iNOS) and cyclooxygenase-2 (COX-2), all of which are key markers of inflammation. MOE treatment, however, effectively reduced these inflammatory markers in a dose-dependent manner. Specifically, MOE suppressed LPS-induced NO production and reduced the expression of iNOS and COX-2, which are enzymes known to contribute to neuroinflammatory damage [17, 18]. These findings are consistent with previous reports showing that *M. officinalis* and its bioactive components, such as rosmarinic acid, have anti-inflammatory effects by inhibiting the production of pro-inflammatory mediator [19]. Furthermore, the gene expression of NLRP3, a key component of the inflammasome complex involved in neuroinflammation, was significantly upregulated in LPS-stimulated BV2 cells. MOE co-treatment notably decreased NLRP3 expression, indicating that MOE may inhibit inflammasome activation, which plays a crucial role in chronic inflammation and neurodegenerative conditions [20-22] . This highlights the potential of MOE to regulate both upstream and downstream inflammatory responses, offering a multifaceted approach to mitigating neuroinflammation.

The MAPK and NF-κB pathways are well-established regulators of inflammatory responses in microglia, and their activation leads to the transcription of pro-inflammatory cytokines and other inflammatory mediators [23-25]. In the current study, LPS-induced phosphorylation of MAPKs, including p38, ERK, and JNK, was significantly reduced by MOE in a concentration-dependent manner. Additionally, NF-κB activity, which was elevated by LPS, was markedly inhibited by MOE co-treatment. This suggests that MOE can effectively downregulate key signaling pathways involved in microglial activation, thereby reducing the production of pro-inflammatory cytokines and preventing further neuronal damage. The ability of MOE to inhibit both MAPK and NF-κB pathways underscores its potential as a neuroprotective agent capable of suppressing multiple inflammatory signaling cascades simultaneously. Oxidative stress, driven by the overproduction of ROS, is a major contributor to neuronal injury and neurodegenerative diseases [26]. In this study, LPS stimulation increased ROS production and led to oxidative damage in BV2 cells, as indicated by the elevated expression of RAGE, a receptor implicated in oxidative stress and inflammation [27-29]. However, MOE treatment significantly reduced ROS levels and downregulated RAGE expression, suggesting that MOE can mitigate LPS-induced oxidative damage. Additionally, MOE enhanced the expression of antioxidant enzymes, including catalase, SOD2 and GLO-1 which play essential roles in detoxifying ROS and protecting cells from oxidative stress. Moreover, MOE increased the nuclear translocation of Nrf2, a transcription factor that regulates the expression of antioxidant genes, and upregulated the expression of HO-1, a downstream target of Nrf2 known for its cytoprotective properties [30-32]. These findings suggest that MOE activates the Nrf2/HO-1 signaling pathway, which is crucial for enhancing the cellular antioxidant response and protecting against oxidative stress. The activation of Nrf2 by MOE further supports its role as a neuroprotective agent, as Nrf2 is a key regulator of cellular defense mechanisms against oxidative damage in the central nervous system In addition, by increasing GLO-1 activity and decreasing RAGE expression, MOE appears to provide a dual mechanism for reducing both oxidative stress and inflammation.

In summary, *M. officinalis* exerts strong anti-inflammatory and antioxidant effects in LPS-stimulated BV2 microglial cells. It suppresses microglial activation by inhibiting key inflammatory signaling pathways, such as MAPK and NF-κB, and enhances antioxidant defense mechanisms through the activation of the Nrf2/HO-1 pathway and the upregulation of catalase and SOD2. Additionally, MOE modulates the activity of GLO1 and reduces RAGE expression, further protecting cells from oxidative damage. These findings suggest that *M. officinalis* may serve as a promising therapeutic agent for the treatment of neuroinflammatory and neurodegenerative diseases. Future experiments should involve animal models to confirm the neuroprotective effects and investigate the molecular mechanisms of individual compounds in MOE.

In this study, rosmarinic acid was selected as the primary marker compound for *M. officinalis* due to its high concentration and well-documented anti-inflammatory properties, including the inhibition of key pro-inflammatory enzymes like COX and 5-LOX [33]. While rosmarinic acid likely contributes significantly to the observed anti-inflammatory effects, other bioactive compounds, such as flavonoids, citral, and geraniol, may also play a role. Further research will explore the individual contributions of these compounds to provide a more comprehensive understanding of the extract’s bioactivity.

## Conclusion

These findings suggest that *M. officinalis* could serve as a promising therapeutic agent for preventing and treating neuroinflammatory and neurodegenerative diseases. By simultaneously targeting inflammation and oxidative stress, MOE offers a multifaceted approach to modulating microglial activation and protecting neurons from damage, positioning it as a potential candidate for further development in neurotherapeutics.

## Figures and Tables

**Table 1:** Analytical results of linearity limit of detection and limit of quantification.

Analyte	Regression equation	Correlation coefficient (R^2^)	Linear range (μg/μl)	LOD^a^ (μg)	LOQ^b^ (μg)
Rosmarinic acid	y = 11624051.21 x – 66398.36	0.999	0.03125 – 1	0.00391	0.01184

LOD^a^ : limit of detection

LOQ^b^ : limit of quantification

**Table 2:** Analytical results of intra-day and inter-day tests.

Analyte	Concentration (μg/μl)	Intra-day			Inter-day		
		Mean (μg/μl)	RSD (%)	Accuracy (%)	Mean (μg/μl)	RSD (%)	Accuracy (%)
Rosmarinic acid	0.125	0.126	0.632	99.823	0.124	0.213	100.225
	0.250	0.252	0.137	99.821	0.251	0.201	99.911
	0.500	0.506	0.531	99.764	0.505	0.542	99.804

RSD : Relative standard deviation

**Table 3:** Contents of major compounds in *M. officinalis* extracts (*n* = 3).

Analyte	Contents (mg/g of dried weight)
Rosmarinic acid	45.51 ± 0.71
